# Cytomegalovirus Proctitis in a Patient with Chronic Lymphocytic Leukemia on Ibrutinib Therapy: A Case Report

**DOI:** 10.7759/cureus.7837

**Published:** 2020-04-26

**Authors:** Yeshaswini Reddy, Muhammad Baig, Nikhil Kalva, Srinivas Puli, Sonu Dhillon

**Affiliations:** 1 Internal Medicine, University of Illinois College of Medicine at Peoria - OSF Saint Francis Medical Center, Peoria, USA; 2 Gastroenterology, University of Illinois College of Medicine at Peoria - OSF Saint Francis Medical Center, Peoria, USA

**Keywords:** cmv proctitis, immunosuppression, ibrutinib

## Abstract

Ibrutinib is a Bruton tyrosine kinase (BTK) inhibitor that has shown significant efficacy in patients with lymphoid carcinomas, mostly chronic lymphocytic leukemia (CLL). Cytomegalovirus (CMV) infection is not a common infectious complication associated with ibrutinib. To increase the clinical awareness about this rare entity, we present the first case of CMV proctitis in an immunocompromised host who was being treated with ibrutinib.

An 88-year old female with a history of CLL treated with ibrutinib presented with two days of painless hematochezia. Physical examination revealed cachexia and temporal wasting; bright red blood was observed on the digital rectal examination. A complete blood count demonstrated a significant decrease in hemoglobin from her baseline. Subsequent colonoscopy revealed a circumferential rectal ulcer; biopsy of the rectal ulcer was positive for CMV immunostain. The patient was treated with intravenous ganciclovir and later transitioned to valganciclovir for a total of 21 days of treatment. Her condition resolved, and she was found doing well at the follow-up visit.

## Introduction

Ibrutinib is used for the treatment of relapsed or refractory chronic lymphocytic leukemia (CLL) in patients who have failed other chemo-immunotherapies [1,2]. The target of ibrutinib is the Bruton tyrosine kinase (BTK), an enzyme that is crucial for B- and T-cell proliferation and survival [3]. However, ibrutinib has been associated with several opportunistic infections, especially in the first 6-12 months of initiation of therapy [3,4]. Cytomegalovirus (CMV) is an opportunistic infection of immunosuppressed hosts due to T-cell dysfunction that occurs due to chronic antigen stimulation in the setting of latent viral infection or underlying cancer [1,5-7]. Gastrointestinal (GI) CMV infection is more pronounced in this patient population and leads to increased overall morbidity and mortality [6,8]. The pathophysiology is due to altered cellular immunity with impaired B- and T-cell function leading to the reactivation of CMV virus [3,7,9]. Here we report the case of an elderly female patient who presented with acute hematochezia and was eventually diagnosed with CMV proctitis in the background of ibrutinib immunosuppressive therapy. This is the first case of CMV proctitis in an immunocompromised host who was being treated with ibrutinib.

## Case presentation

An 88-year-old Caucasian female presented to the emergency room with a two-day history of painless hematochezia with associated generalized fatigue and unintentional weight loss of 10 pounds. She denied any change in her bowel habits, abdominal or rectal pain, fever, nausea, or vomiting episodes. Her past medical history was significant for CLL being treated with oral ibrutinib 420 mg daily. She was hemodynamically stable and physical examination revealed cachexia and temporal muscle wasting; bright red blood was observed on the digital rectal examination. At this point, our differential diagnosis included hemorrhage secondary to diverticulosis, colorectal angiodysplasias, or internal hemorrhoids.

**Figure 1:**
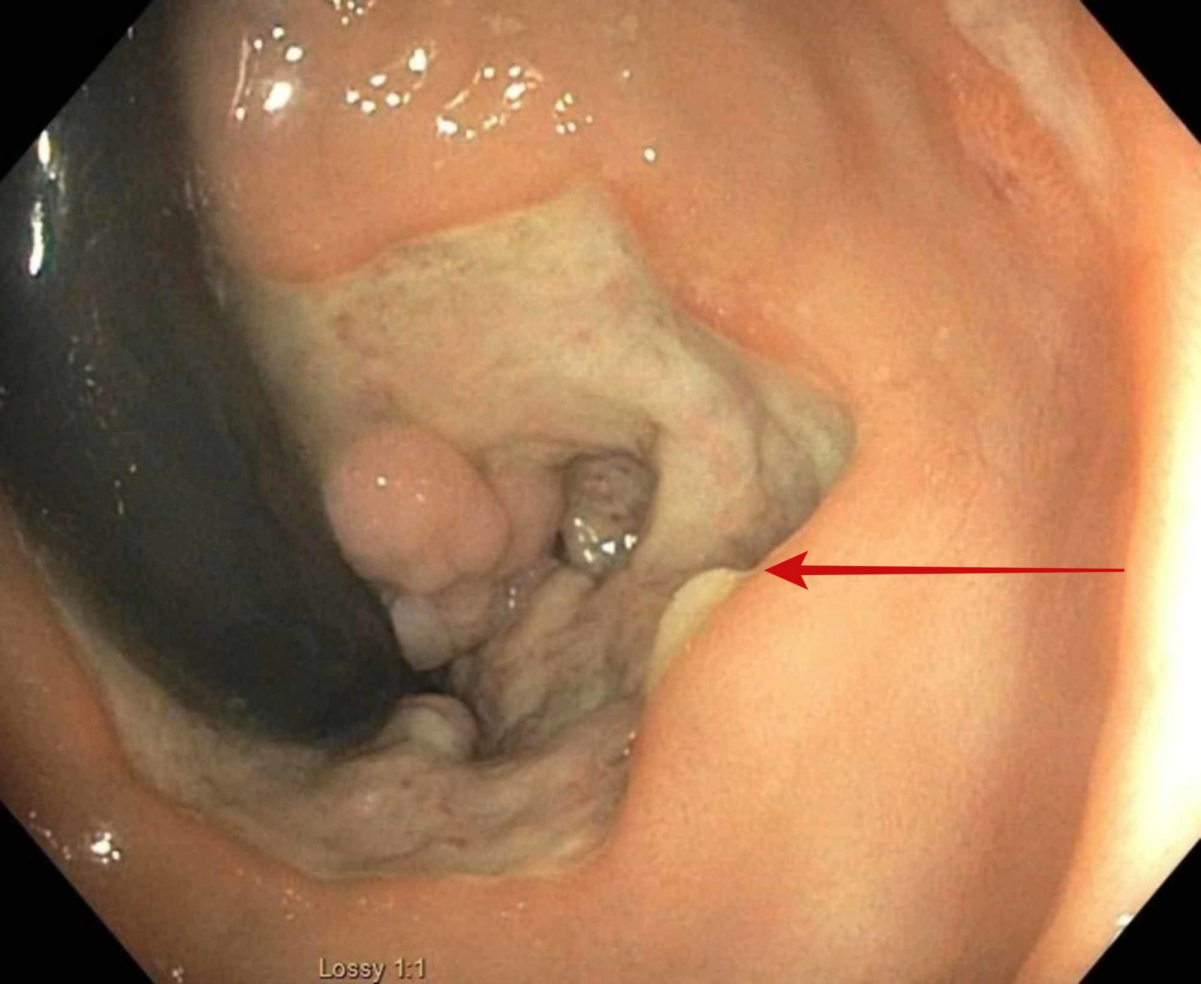
Retroflexed view of rectum showing a circumferential rectal ulcer (red arrow)

**Figure 2:**
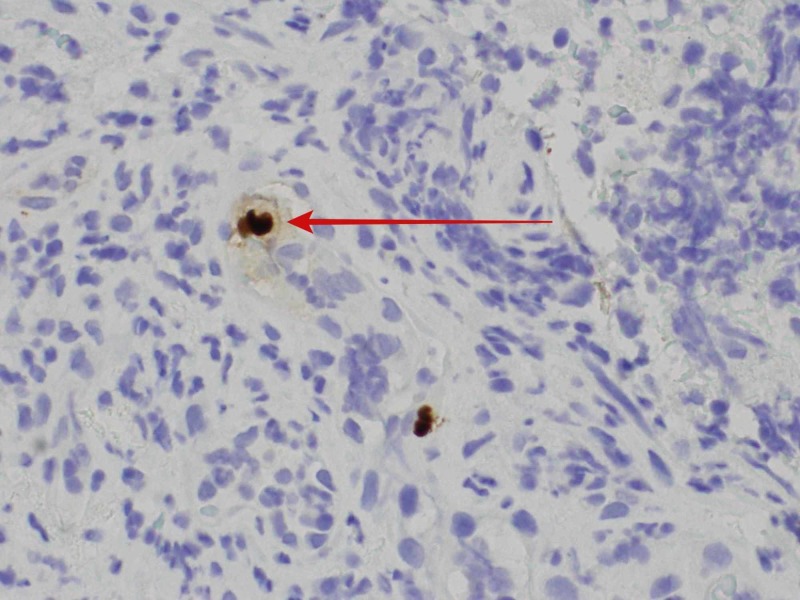
Immunostaining positive for CMV in the rectal ulcer with an illustration of an “owl eye inclusion” (red arrow) CMV: cytomegalovirus

The following investigations were normal or negative: basic metabolic panel, coagulation profile, urinalysis, and chest X-ray. A complete blood count showed a reduction in hemoglobin level to 8.1 g/dL compared to a baseline of 11.5 g/dL. Further evaluation with colonoscopy revealed a circumferential, deep, and clean-based rectal ulcer in close proximity to the dentate line (Figure 1). The rest of the colorectal mucosa was noted to be unremarkable. Biopsy of the rectal ulcer showed superficial fragments of ulcerated granulation tissue with no definitive viral cytopathic changes on hematoxylin and eosin stain. However, CMV immunostain was positive (Figure 2). Serum polymerase chain reaction (PCR) to detect CMV DNA was negative for viremia.

The patient was diagnosed with CMV proctitis secondary to immunodeficiency from ibrutinib treatment. She was initially started on intravenous ganciclovir 5 mg/kg twice daily for five days and then transitioned to oral valganciclovir 900 mg twice daily for a total of 21 days of treatment. Her hematochezia gradually resolved in three days and she appeared to be doing well at her follow-up visit.

## Discussion

Ibrutinib inhibits the BTK, which is an important signaling molecule in the pathogenesis of CLL, and has proven to suppress the B-cell lymphoproliferation and induce apoptosis of CLL cells [1,2]. Patients treated with BTK inhibitors are at increased risk of developing hypo-gammaglobulinemia due to the impairment of humoral immunity [1,2,4]. Several studies have also shown that ibrutinib decreases the regulatory T-cells by decreasing Th-2 cytokines in the cells during the first 6-12 months after initiation of therapy, thereby increasing the risk of opportunistic infections during this time period [10,11]. The most common infections associated with this drug are respiratory tract infections, followed by skin and ear infections [1,4].

CMV is a latent herpes virus infection that can undergo reactivation in an immunosuppressed host, leading to an increased risk of mortality and morbidity [5,6,9]. Therefore, early diagnosis and initiation of antiviral therapy are crucial for improved outcomes [5,9,10]. Patients with hematological neoplasms have impaired T-cell function, which increases their risk of CMV reactivation [9]. These patients, when started on immunosuppressant medications like ibrutinib, which alter the cellular immunity by further suppressing B- and T-cell function, are at further increased risk for CMV infections [3,9]. In the GI tract, CMV infection may appear from mouth to anus, and tissue necrosis is noted to be a prominent feature [6,10]. Diagnosis is usually made by visualizing the large cells with intra-nuclear and intra-cytoplasmic inclusions as seen in our case. Despite the normal serological testing, colonoscopy and biopsies should be considered for diagnostic confirmation. Ibrutinib should be withheld in patients hospitalized with any infection and can be restarted once the infection is resolved. There are multiple drug interactions associated with ibrutinib therapy, and the literature does not support prophylactic antiviral therapy to prevent recurrent CMV infection [11].

## Conclusions

CMV proctitis is an opportunistic infection that is commonly seen in immunosuppressed patients. These patients usually present with symptoms mimicking gastroenteritis or painless GI blood loss due to mucosal ulcerations. With the increasing utilization of immunosuppressive therapies like ibrutinib, CMV infections should be considered in the right clinical setting. Early diagnosis and treatment with appropriate antiviral therapy are critical for good outcomes. Further studies are required to investigate the benefits of empiric antiviral therapy in this patient population.
